# Unsupervised machine learning using an imaging mass spectrometry dataset automatically reassembles grey and white matter

**DOI:** 10.1038/s41598-019-49819-1

**Published:** 2019-09-13

**Authors:** Makoto Nampei, Makoto Horikawa, Keisuke Ishizu, Fumiyoshi Yamazaki, Hidemoto Yamada, Tomoaki Kahyo, Mitsutoshi Setou

**Affiliations:** 1 Department of Cellular and Molecular Anatomy, Hamamatsu University School of Medicine, Hamamatsu, Shizuoka 431-3192 Japan; 2 International Mass Imaging Center, Hamamatsu University School of Medicine, Hamamatsu, Shizuoka 431-3192 Japan; 3 Department of Systems Molecular Anatomy, Institute for Medical Photonics Research, Preeminent Medical Photonics Education & Research Center, Hamamatsu University School of Medicine, Hamamatsu, Shizuoka 431-3192 Japan

**Keywords:** Imaging, Classification and taxonomy, Structural biology

## Abstract

Current histological and anatomical analysis techniques, including fluorescence *in situ* hybridisation, immunohistochemistry, immunofluorescence, immunoelectron microscopy and fluorescent fusion protein, have revealed great distribution diversity of mRNA and proteins in the brain. However, the distributional pattern of small biomolecules, such as lipids, remains unclear. To this end, we have developed and optimised imaging mass spectrometry (IMS), a combined technique incorporating mass spectrometry and microscopy, which is capable of comprehensively visualising biomolecule distribution. We demonstrated the differential distribution of phospholipids throughout the cell body and axon of neuronal cells using IMS analysis. In this study, we used solarix XR, a high mass resolution and highly sensitive MALDI-FT-ICR-MS capable of detecting higher number of molecules than conventional MALDI-TOF-MS instruments, to create a molecular distribution dataset. We examined the diversity of biomolecule distribution in rat brains using IMS and hypothesised that unsupervised machine learning reconstructs brain structures such as the grey and white matters. We have demonstrated that principal component analysis (PCA) can reassemble the grey and white matters without assigning brain anatomical regions. Hierarchical clustering allowed us to classify the 10 groups of observed molecules according to their distributions. Furthermore, the group of molecules specifically localised in the cerebellar cortex was estimated to be composed of phospholipids.

## Introduction

The mammalian brain is composed of a multitude of cell types, and its spatial structure is commensurately highly complex. Regions of the brain have been distinguished and segmented using several criteria, including macro- and micro-scale anatomical and neurological measures^1–6^. Recent molecular anatomical approaches, such as fluorescence *in situ* hybridisation, immunohistochemistry, immunofluorescence, immunoelectron microscopy and fluorescent fusion protein, have comprehensively distinguished the distributions of mRNA^7–12^ and proteins^13–15^. Molecular biological approaches, such as Brainbow, have the potential to demonstrate the distributions of different neuronal cells in animal brains^16,17^. The brain-mapping project could also show both fibre structures and neuronal networks of the whole brain^18–20^. At present, several brain atlases are available, including those of mice, rats and humans (Allen Brain Atlas, http://portal.brain-map.org/; Human Protein Atlas, https://www.proteinatlas.org/)^21–26^. Although these atlases can display distributions of mRNA and proteins in the brain, the distributions of biomolecules, such as lipids, carbohydrates and amino acids, in the brain remain unclear.

The distributions of biomolecules in the brain can be analysed using several techniques. Among these are radioisotope and fluorescent tagging, with applicable spatial resolutions ranging between those of optical microscopy and electron microscopy, as well as techniques relating to mRNA and proteins^27–30^. However, since some metabolic processes convert labelled molecules into other forms, fluorescent tags can change the biological nature of molecules and immunostaining is applicable only to a restricted range of biomolecules, these techniques are unsuited to the observation of most biomolecule distributions. Mass spectrometry has the potential to comprehensively analyse a wider range of biomolecules. Through a combination of histological techniques, e.g. laser microdissection and micro-extraction, mass spectrometry can analyse tissue-specific compositions of biomolecules^31,32^. These approaches have high detection sensitivity of biomolecules, but their spatial resolutions are several hundred micrometres. To investigate the distributions of biomolecules in the brain with a high spatial resolution like optic microscopy, an alternative technique is required.

Imaging mass spectrometry (IMS), integrating mass spectrometry and microscopy, can visualize the distribution of biomolecules at a spatial resolution of a few micrometres^33^. Using the technique, we have observed the distribution of biomolecules, especially lipid species, in the brain, both with and without pathological changes and revealed that several biomolecules had distinct distributions^34–37^. We also demonstrated a gradient distribution of axon phospholipid species^38,39^ and the polarity of phospholipid delivery^40^. These findings showed the differential distribution of lipid species in cell bodies and neuronal fibres, namely in the white and grey matters. Conversely, we recently demonstrated that the compositions of biomolecules are highly similar in different areas of the same tissues and even within different organs^41^.

These previous findings led us to the possibility that it can classify the distributions of biomolecules in the brain at least as three groups: in white matter, in grey matter and homogeneously expressed. Pattern analysis, one of unsupervised machine learning techniques, can automatically and unbiasedly classify molecules with their properties^42,43^. We hypothesise that principal component analysis (PCA) was able to reconstruct the distributions of molecules localised in the grey and white matters. Furthermore, using hierarchical clustering, we have discovered a novel group of molecules distributed in a specific region. Here, we examine the diversity of biomolecule distribution in the brain using IMS and unsupervised machine learning techniques. For the IMS analysis, we selected dihydroxybenzoic acid (DHB), a widely used Matrix Assisted Laser Desorption/Ionisation (MALDI) matrix, for the ionisation of phospholipids^34^. An IMS dataset was constructed using solarix XR, the most recent MALDI-IMS instrument, installed on a Fourier transform ion cyclotron resonance mass spectrometer (FT-ICR-MS), with a high molecular sensitivity and mass resolution^44,45^. Using such an instrument, IMS can measure the distributions of many more molecules compared with the previous generation of IMS instruments, such as time-of-flight mass spectrometry (TOF-MS). Finally, we challenged to investigate the diversity of molecule distributions in the brain.

## Results

### MALDI-IMS analysis of the rat brain section was conducted using MALDI-FT-ICR-MS

**Figure 1:**
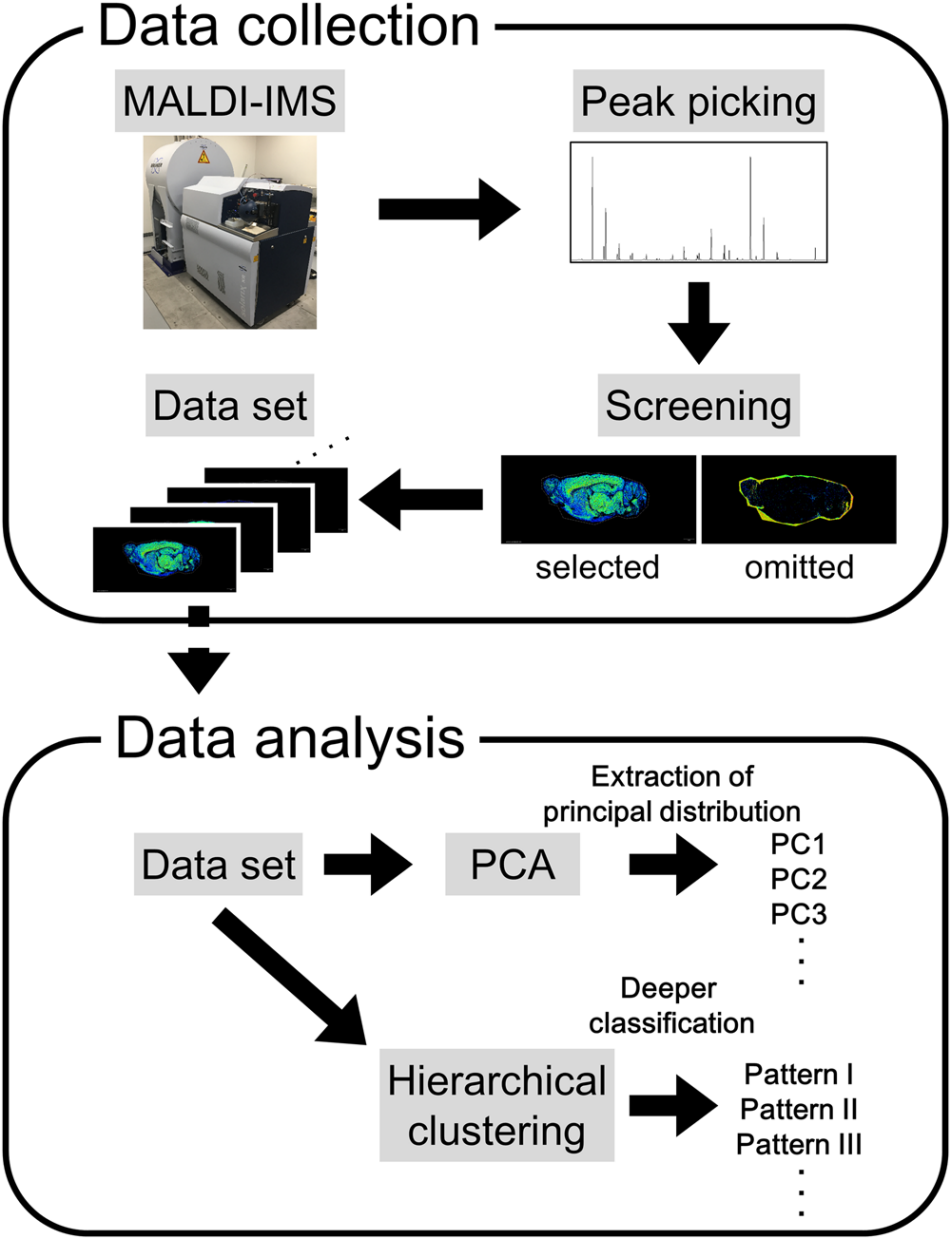
Schematic image of IMS data collection and analysis. The data collection has four steps: MALDI-IMS of the sagittal section of a rat brain, peak picking from the mass spectrum, screening of the distributions of biomolecules and the construction of an IMS dataset. Data analysis was undertaken by performing PCA to extract the principal distribution from the IMS dataset. Hierarchical clustering was used to classify molecules by their patterns of distribution.

**Figure 2:**
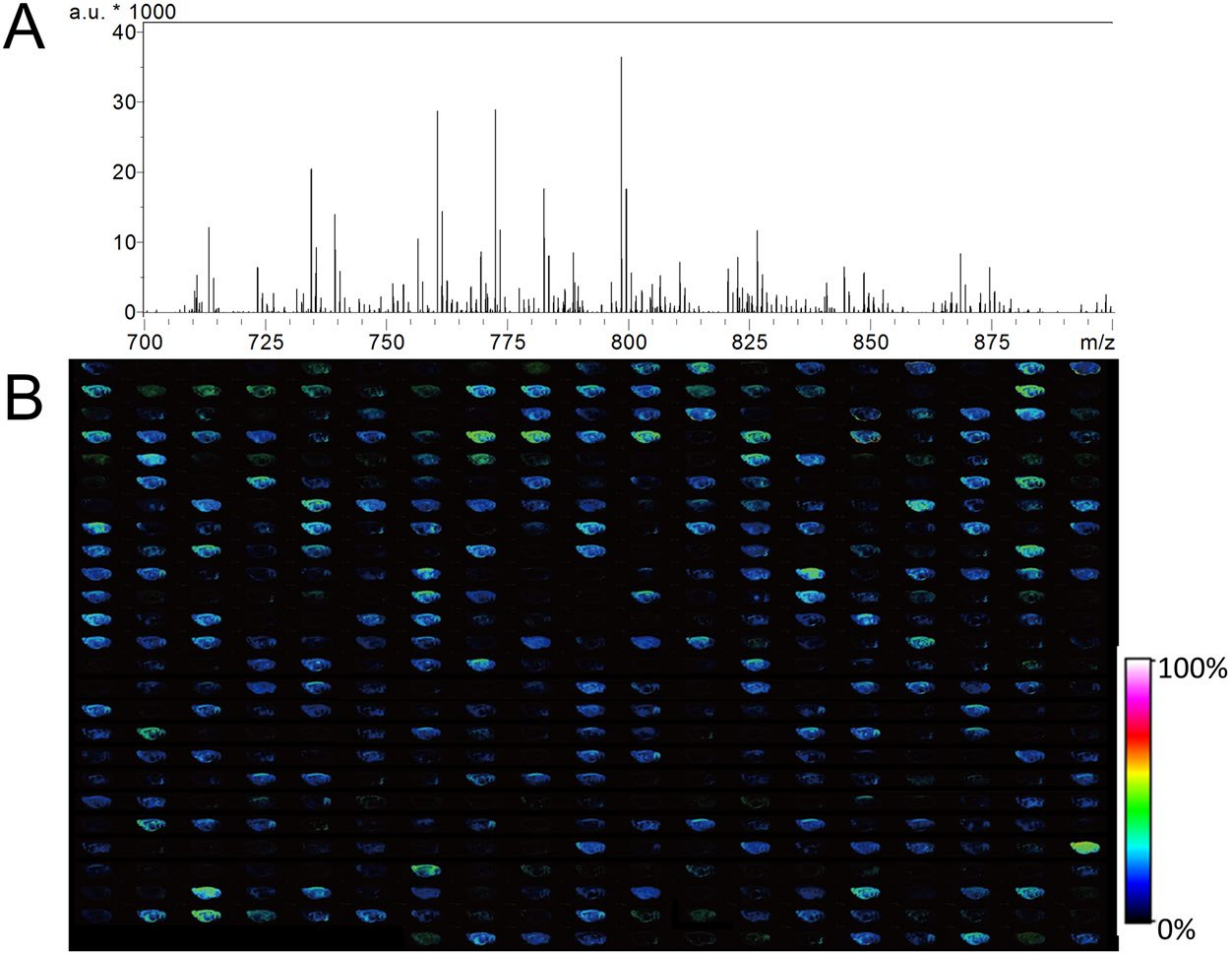
Data collection from rat brain sagittal sections using MALDI-FT-ICR-IMS. (**A**) Average mass spectrum of all measured points. The x-axis corresponds to the mass range between *m/z* 700 to 900, whereas the y-axis shows relative signal intensity. a.u., arbitrary unit. (**B**) Lineup of all biomolecule distributions from a sagittal section of a rat brain in an IMS dataset, for which the number of distributions was 488. The signal intensity of each spot is shown using a rainbow scale.

MALDI-IMS analyses were conducted using a solarix XR MALDI-IMS instrument furnished with FT-ICR-MS. Mass spectra were obtained at multiple points from the sagittal section of rat brains. After the acquisition, we performed peak picking in the mass range from 700 to 900 using fleximaging 4.1 software. For the obtained peaks, we manually selected distributions derived from biomolecules and output the dataset. We assigned distributions overlapping with the brain region as biomolecules and excluded that were detected homogeneously on the brain and the slide or had higher intensities outside of the brain as artefacts. Dataset construction and analysis procedures are summarised in Fig. 1. MALDI-IMS analysis allowed us to obtain a mass spectrum of 55,495 points from the sagittal section of rat brains (Fig. 2A). After the manual selection, we acquired a dataset containing 488 distributions derived from biomolecules (Fig. 2B).

### Intensity distribution of the PC1 reassembly of grey and white matter using PCA

**Figure 3:**
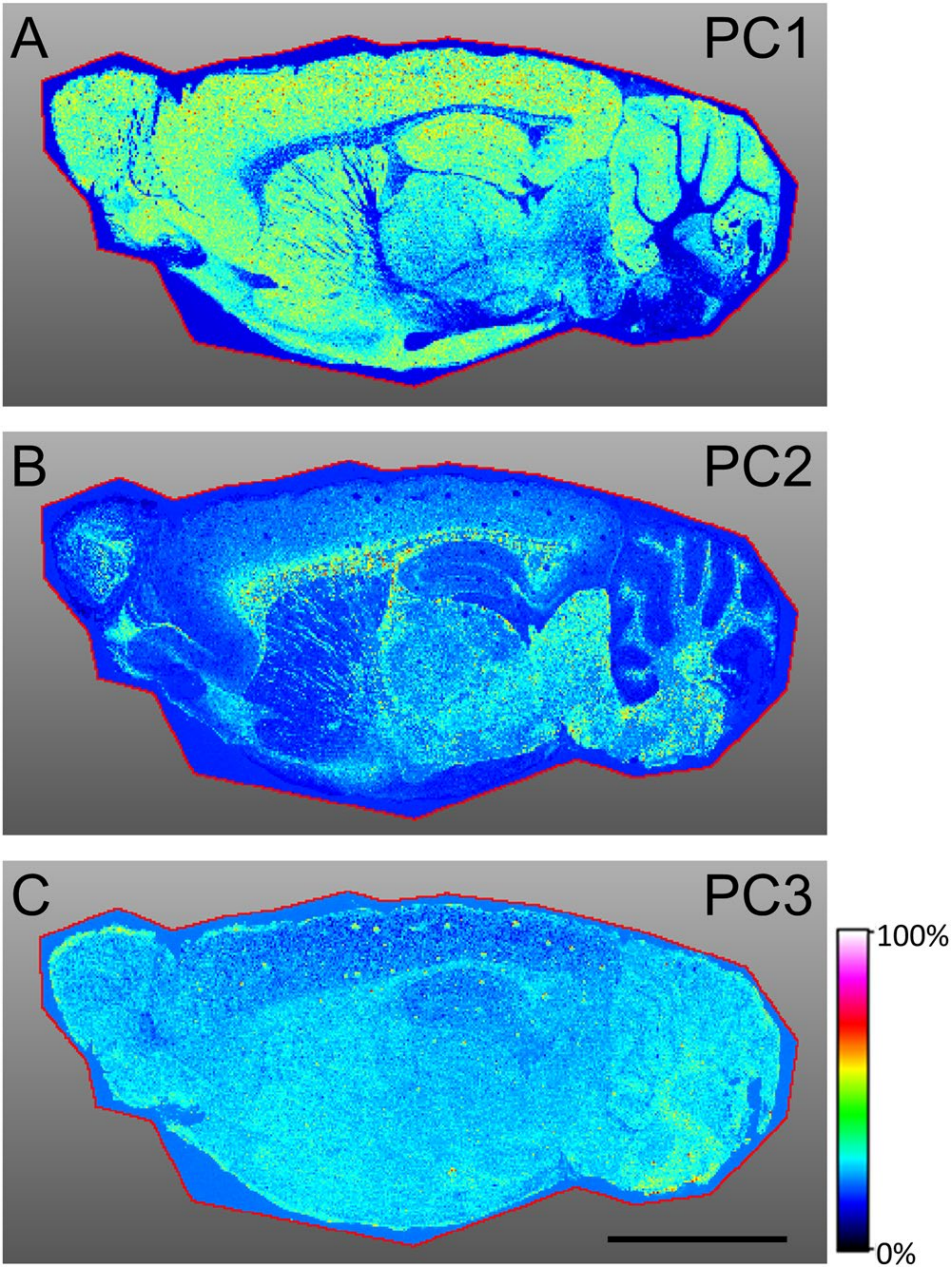
PCA of the sagittal section of a rat brain. Rainbow scale, intensity distributions of (**A**) PC1, (**B**) PC2 and (**C**) PC3 were displayed using a rainbow colour scale. The red line indicates the border of the region of interest (ROI). The area of ROI surrounded the whole brain section. Black scale bar = 5 mm.

Using the IMS dataset, we performed PCA and reassembled the distribution of biomolecules using SCiLS Lab software. This showed that the distribution of PC1 chiefly overlapped with the regions corresponding to grey matter: the cerebral cortex, cerebellar cortex, interbrain, midbrain, olfactory bulb, striatum, hippocampus and hypothalamus (Fig. 3A). On the other hand, PC2 was mostly observed in fibre tracts: corpus callosum, fornix, stria terminalis, internal capsule, olfactory nerve layer, lateral olfactory tract, cerebral peduncle, arbor vitae, pons and medulla (Fig. 3B). The distribution of PC2 also overlapped with the interbrain, midbrain and hindbrain. PC3 was found to be almost uniformly present throughout the brain, albeit with slightly lesser intensity in the cerebral cortex and a stronger intensity in the medulla (Fig. 3C). These intensity distributions indicate that there are at least three major distributions of biomolecules in rat brains. The Contributions of PC1, PC2 and PC3 were 19.8% 9.1% and 6.0%, respectively.

### Pattern recognition using hierarchical clustering analysis

**Figure 4:**
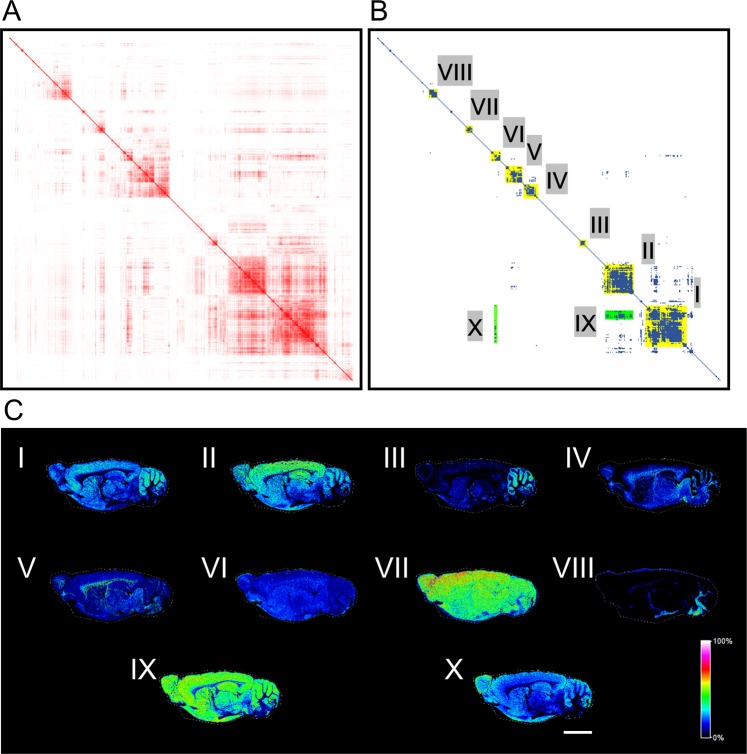
Hierarchical clustering of an IMS dataset. Heat maps showing (**A**) the relative values of cosine similarities in red colour gradient and (**B**) spots having the values larger than 0.5 in blue. Groups consisting of more than seven biomolecules on the diagonal line were marked in yellow (I–VIII) and those of out of the line in green. (**C**) Representative distributions of each group (I to X). The rainbow colour scale reflects the signal intensity of each sampling point. Scale bar = 5 mm.

To classify these three major distribution patterns in detail, we performed hierarchical clustering analyses using the same IMS dataset. SCiLS Lab software was used to produce heat maps with similarity values (Fig. 4A and Table S1). The diagonal line indicates combinations where the distributions have the same *m/z*. Following this, we selected points denoting higher similarity between pairs of distributions on the heat map using a threshold similarity value of 0.5 and obtained eight distributional groups (I–VIII) on the diagonal line and two groups (IX and X) out of the line (Fig. 4B). We found that these groups had distinct distributions (Fig. 4C and Table S2). The occupancies of each group in the total distributions are listed in Table 1.

**Table 1:** The number and the ratio of molecules in each group out of the total molecules in the hierarchical clustering.

Group	Number	Ratio (%)
I	59	12.1
II	43	8.8
III	7	1.4
IV	19	3.9
V	23	4.7
VI	13	2.7
VII	9	1.8
VIII	11	2.3
Grouped total	184	37.7

Of these, group **I** was the largest, primarily localised in the cerebral cortex, cerebellar cortex, interbrain, midbrain, olfactory bulb, striatum, hippocampus and hypothalamus; its distributional pattern was similar to that of PC1 of PCA (Figs 3A and 4C). Group **II** was the second largest, with a distribution similar to that of group **I** and PC1 (Figs 3A and 4C); however, the signals present in the cerebellar cortex were slightly higher than other regions in group **I**, whereas the cerebral cortex showed the stronger signal intensity in group **II**. Groups **IX and X** also displayed similar distributions to PC1, but their signal intensities, where detected, were homogeneous (Figs 3A and 4C). Group **III** was chiefly observed in the cerebellar cortex (Fig. 4C). No signals were observed in the fibre tracts in groups **I, II, III, IX and X** (Fig. 4C) although, contrarily, we detected significant fibre tract signals for groups **IV and VIII** (Figs 3B and 4C). In group **VIII**, signals were detected in the corpus callosum, fornix, internal capsule, cerebral peduncle, arbor vitae, pons and medulla (Fig. 4C). The distribution of group **IV** resembled PC2, being located in the fibre tracts (corpus callosum, fornix, stria terminalis, internal capsule, olfactory nerve layer, lateral olfactory tract, cerebral peduncle, arbor vitae, pons and medulla), as well as in the interbrain, midbrain and hindbrain (Figs 3B and 4C). Signals were observed throughout the brain, but strongly detected in fibre tracts in group **V** (Fig. 4C). In groups **VI and VII**, homogeneous signal intensities were detected throughout the brain section as with PC3 (Figs 3C and 4C). Group **VII** showed high signal intensity in the cerebral cortex compared to other regions (Fig. 4C).

### Molecules highly distributed in the cerebellar cortex were estimated to be phospholipids

**Figure 5:**
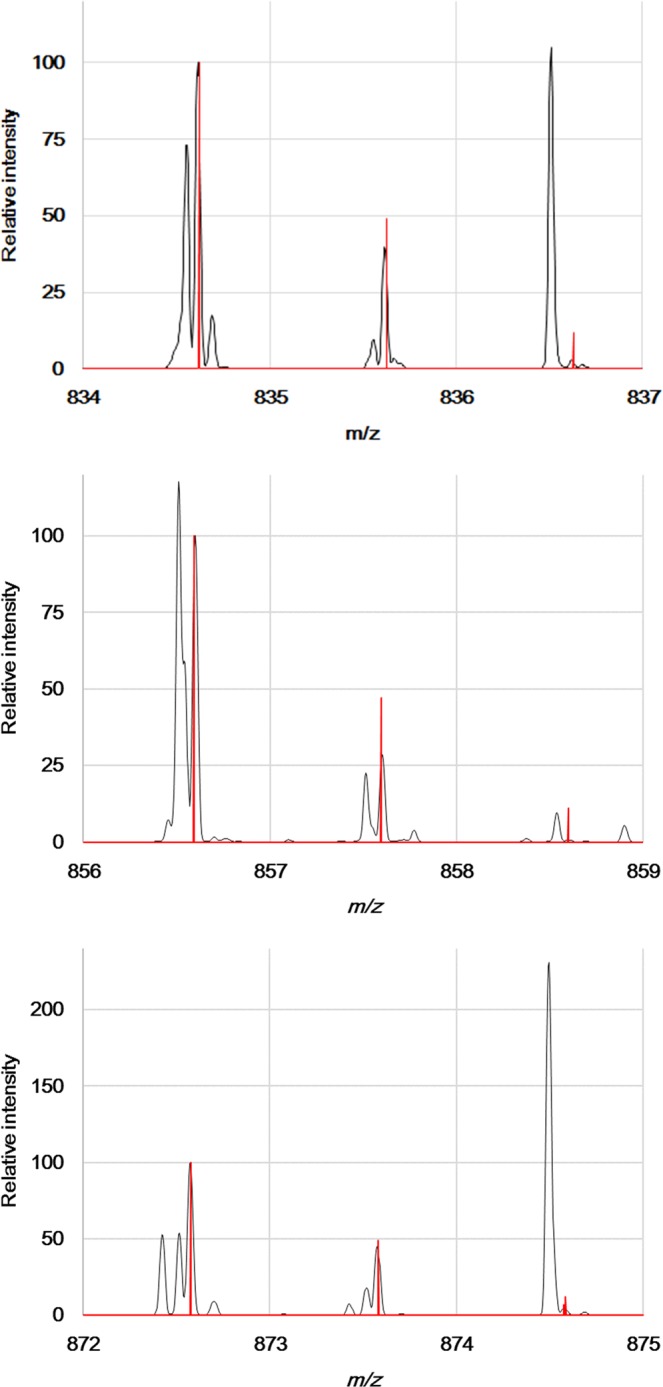
The comparison of observed mass spectra and the calculated isotope pattern. The graphs showing mass spectra from *m/z* 834 to 837, *m/z* 856 to 859, and *m/z* 872 to 875 (black lines). The red lines indicated the calculated isotope patterns of [PS(39:0) + H]^+^, [PI(34:0) + NH4]^+^ and [PS(39:0) + K]^+^.

**Figure 6:**
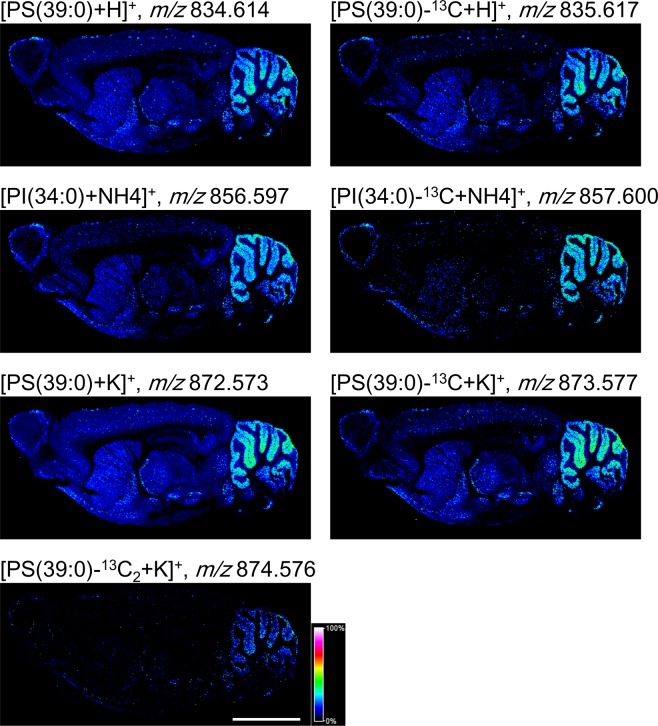
The molecular group distributed within the cerebellar cortex. Representative distributions of molecules belonging to group III. The rainbow colour scale reflects the signal intensity of each sampling point. Scale bar = 5 mm.

Hierarchical clustering found a group of molecules that were highly expressed in the cerebellar cortex (Fig. 4C, group III). The group C contained seven molecular distributions and some of them exhibited mass differences between *m/z* values of 1.0 Da: *m/z* 834.614 and *m/z* 835.617, *m/z* 874.576 and *m/z* 857.600, and *m/z* 872.573, *m/z* 873.577 and *m/z* 874.576. We then hypothesized the mass differences were derived from ^13^C-isotopes and calculated isotope patterns of these molecules. At first, we estimated the *m/z* values of the smaller ions using the Human Metabolome Database and assigned *m/z* 834.614 to [PS(39:0) + H]^+^, *m/z* 856.597 to [PI(34:0) + NH4]^+^ and *m/z* 872.573 to [PS(39:0) + K]^+^. The observed mass spectra were well fitted to the relative intensities of the calculated isotope patterns (Fig. 5 and Table 2). The results demonstrated that *m/z* 835.617 was ^13^C-isotope of [PS(39:0) + H]^+^, *m/z* 857.600 was that of PI(34:0) + NH4]^+^, and *m/z* 873.577 and *m/z* 874.576 were that of [PS(39:0) + K]^+^. It suggests that the group C was just composed of isotopes of two phospholipids with different adducts.(Fig. 6). We showed the theoretical and observed masses of the phospholipids and their isotopes with the mass errors in Table 3.

**Table 2:** Relative intensities and mass differences of the molecules in the group C.

m/z	Relative intensity	Difference in mass from a lighter molecule	m/z	Relative intensity	Difference in mass from a lighter molecule
(observed)	(observed)	(observed)	(theoretical)	(theoretical)	(theoretical)
834.614	100	1.003	834.6225	100	1.0034
835.617	40		835.6258	49	1.0034
			836.6292	12	
856.597	100	1.003	856.5916	100	1.0034
857.600	28		857.5949	47	1.0034
			858.5983	11	
872.573	100	1.004	872.5783	100	1.0034
873.577	44	0.999	873.5817	49	0.9948
874.576	4		874.5765	7	0.0086
			874.5851	12

**Table 3:** Assignment of ions distributed throughout the cerebellar cortex.

*m/z* (observed)	Estimated molecule	Theoretical mass	Adduct	*m/z* (adduct)	delta ppm
834.614	PS(39:0)	833.6146	[M + H]^+^	834.6224	−10.06
835.617	PS(39:0) isotopic	834.6179	[M + H]^+^	835.6258	−10.53
856.597	PI(34:0)	838.5571	[M + NH4]^+^	856.5915	6.42
857.600	PI(34:0) isotopic	839.5605	[M + NH4]^+^	857.5949	5.95
872.573	PS(39:0)	833.6146	[M+K]^+^	872.5783	−6.07
873.577	PS(39:0) isotopic	834.6179	[M+K]^+^	873.5816	−5.27
874.576	PS(39:0) isotopic	835.6213	[M+K]^+^	874.5850	−10.29

## Discussion

In this study, we developed a novel approach for IMS data analysis that produces an unbiased reconstruction of tissue structure using a large IMS dataset. We demonstrated that typical brain structures could be reassembled using PCA; PC1 corresponded to grey matter and PC2 to fibre tracts. This result suggests that, despite the diversity of molecular distribution in the brain, its structure can be reconstructed using PCA. Furthermore, PC3 showed an almost homogeneous distribution throughout the studied brain sections, indicating that many biomolecules in the brain have endemic distributions. In previous IMS studies, we observed that phosphatidylcholine(PC) species showed own distinct distributions expressed in white matter, grey matter and ubiquitously^36,46^, but we could not determine which pattern of distribution was greatly observed in the brain with a small number of biomolecule distributions. In this study, we revealed that the number of molecules enriched in grey or white matters were greater than that of molecules expressed homogeneously.

Hierarchical clustering allowed the distributional patterns of biomolecules to be classified at a finer resolution. Therein, groups I, II and III showed very similar signal distributions, but different signal intensities in each region of the brain. Because the molecules in groups I, II and III were expressed in regions corresponding to grey matter, we consider that these molecules are produced or stored in the cell body, such as neurotransmitters^47^. The molecules of group III were mostly distributed in the cerebellar cortex and were estimated to be phospholipids using the *m/z* values of ions from the Human Metabolome Database. We hypothesise that these phospholipids have an important role in the region, but their functions in the cerebellar cortex have not been completely investigated. The molecules classified into groups IV and V were found to spatially associate with white matter, suggesting that they are delivered to fibre tracts from grey matter or synthesised in glial cells or directly in the axon. To elucidate this, we attempted to assign obtained *m/z* values to various molecules. The molecules in group IV were detected in the interbrain, midbrain, hindbrains, pons and medulla, whereas those in group V were observed with weak signal intensities but throughout the brain. It, therefore, seems that the molecules classified into group IV may be synthesised in the brain stem and those from group V are characteristic of the whole brain. The molecules in groups VI and VII were expressed throughout the brain, but the signal intensities of group VII were stronger in the cerebral cortex than in other regions. We believe that this high signal intensity may reflect the localities where the molecules in group VII are synthesised and stored. It is also possible that these molecules are synthesised in regions showing high signal intensity and then dispersed throughout the brain. The ubiquitous expression of these molecules probably implies that they are essential for cellular survival. Contrary to our expectations, we obtained distributional patterns that showed the highest signal intensities in the cerebellar cortex and medulla but could not observe any molecules showing signals specifically in the cerebral cortex, interbrain, midbrain, olfactory bulb, striatum, hippocampus, hypothalamus, pons, or arbor vitae. This suggests either that there are no such molecules or that their numbers are very small. If the criteria for molecular grouping were changed, we would possibly detect molecule groups distributed in these specific brain regions.

As reported in several studies including the present study, IMS is a powerful tool to observe distributions of several small molecules^33–46,48–55^. Compared with IMS instruments equipped with TOF-MS, FT-ICR-IMS is highly sensitive, can distinguish molecules with very similar molecular weights and, therefore, is able to collect a large number of biomolecule distributions^44,45,51^. As demonstrated, MALDI-FT-ICR-IMS could collect approximate 500 distributions in a range of *m/z* 700–900, and the number was large enough to perform clustering analysis. Notably, we found phospholipid species showing high expressions specifically in the cerebellar cortex. However, we obtained duplications of distributions derived from the ^13^C-isotopes and the same molecules with different adducts. Based on Fig. 5, we estimate that approximately half of the distributions may come from ^13^C-isotopes. If we exclude these duplications, the number of distributions in each group may drop down, but the ratio will not change. Furthermore, we think some extra steps would be required to reduce such duplications, for example, ammonium sulfate pre-treatment to samples as we previously reported^34^, and post data analysis to remove distributions of isotopes. In conclusion, we believe that the combination of MALDI-FT-ICR-IMS and unsupervised machine learning techniques is a rapid approach to unbiasedly and automatically classify molecules based on distribution and to easily identify novel molecular groups localised in specific regions.

In the future, similar analyses should be performed with IMS datasets collected using other matrices and different mass ranges^33,49,56^. Such comparative analyses can provide further patterns of molecular distributions. In addition, LC-MS/MS analysis should be conducted to assign the detected *m/z* to specific molecules and to identify the molecules classified into each group. Identification of molecules could facilitate elucidation of their interrelationships. For instance, we can investigate whether the molecules are in the same metabolic pathways, belong to the same molecular classes and have the same biological functions. To accelerate and automate the data collection process, we plan to apply unsupervised machine learning for screening biomolecule distributions and excluding distributions of isotopes. A further potential research direction involves the investigation of the functions of identified molecules combining their distribution with the knowledge of neuronal anatomy.

## Materials and Methods

### Animals

All experimental procedures were approved by the Ethics Committee of the Hamamatsu University School of Medicine (the ethical number for the animal experiment: #2017083) and carried out in accordance with the approved guidelines. Wister male rats aged 8 weeks were purchased from Japan SLC (Hamamatsu, Japan).

### Chemicals and materials

Methanol, diethyl ether and ultrapure water were purchased from Wako Pure Chemical Industries (Osaka, Japan). Sodium trifluoroacetate, used as the calibration standard, was purchased from Sigma-Aldrich Japan (Meguro, Japan)^51^. A MALDI matrix, DHB, was purchased from Bruker Daltonics (Fremont, CA, USA)^53^, whereas 100 Ω indium tin oxide (ITO)-coated glass slides were purchased from Matsunami Glass Ind., Ltd. (Osaka, Japan)^55^.

### Sample preparation

All rats were anaesthetised by diethyl ether before whole brain samples were quick-frozen with powdered dry-ice. These brain samples were stored at −80 °C prior to making sections. The sagittal sections of the frozen brains were sectioned with a thickness of 10 μm at −20 °C using a Cryostat (CM1950, Leica Microsystems K.K., Tokyo, Japan), and the resulting slices were mounted onto ITO glass slides. The matrix was applied onto slides as a 1.0-μm thick layer by sublimation using iMLayer (Shimadzu Corporation, Kyoto, Japan).

### MALDI-IMS analysis

All brain sections were analysed using solarix XR, FT-ICR-IMS (Bruker Daltonics) with an *m/z* range of 650–1150. The laser diameter was set at the smallest value (~25 μm), laser spot raster at 50 μm, laser shot count at 200, time of flight to 0.7 ms and the positive mode was used. Sodium trifluoroacetate (TFA, [M + Na]^+^) was used for the external calibration of FT-ICR-MS^57^.

### Data analysis

We obtained mass spectrum data from each measuring point through the brain sections by MALDI-IMS analyses. Data analysis was performed using fleximaging 4.1 (Bruker Daltonics)^51^ and SCiLS Lab (version 2015b, SCiLs, Bremen, Germany)^54^. Peak picking used a signal-to-noise threshold of 10 and a width of 0.2 Da. To select the distribution patterns of biomolecules, we manually judged signals that showed higher intensities on the brain section as biomolecules. We performed PCA using the following parameters: algorithm, PCA; denoising, None; interval width, ±0.004 Da; interval processing mode, maximum; normalisation, none; number of components, 5; scaling: unit variance and spectrum mode: individual. Hierarchical clustering was performed using *m/z* image generation in SCiLS Lab for all screened distributions to obtain a *m/z* correlation plot. The anatomical position of the rat brain was judged using *the Rat Brain in Stereotaxic Coordinates*^58^ and Rat Brain Atlas (http://labs.gaidi.ca/rat-brain-atlas/). For assignment of molecules, we performed LC-MS searches in the Human Metabolome Database (http://www.hmdb.ca/) using the following criteria: positive ion mode, adduct type [M + H]^+^, [M + NH_4_]^+^, [M + Na]^+^ and [M + K]^+^] and the threshold of 10 ppm.

## Supplementary information

Supplementary information

TableS1

TableS2
