# Supplementary material for: Unsupervised machine learning using an imaging mass spectrometry dataset automatically reassembles grey and white matter
Source: Sci Rep. 2019 Sep 13;9:13213. doi: 10.1038/s41598-019-49819-1 (PMC6744563; doi:10.1038/s41598-019-49819-1)
Supplement: Supplementary file 1 — Supplementary information [file 41598_2019_49819_MOESM1_ESM.docx]

**Title:** **Unsupervised machine learning using an imaging mass spectrometry dataset automatically reassembles grey and white matter**

Makoto Nampei^+,1^, Makoto Horikawa^+,1,2^, Keisuke Ishizu^1^, Fumiyoshi Yamazaki^1,2^, Hidemoto Yamada^1^, Tomoaki Kahyo^,1,2^ and Mitsutoshi Setou^1,2,3,*^

**Table S1 The heat map with hierarchical clustering**

Each spot displayed a similarity value of a pair of molecules. The red colour gradients reflected relative values of similarities of pairs. The *m/z* values of molecules were shown in the top line and the left end column.

**Table S2 The list of *m/z* in the group I to VIII and out groups**

The colours indicated each group. The out-groups were displayed without colour. The m/z values were sorted in order of the result of hierarchical clustering.
